# Free Radical Scavenging, Redox Balance and Wound Healing Activity of Bioactive Peptides Derived from Proteinase K-Assisted Hydrolysis of *Hypophthalmichthys molitrix* Skin Collagen

**DOI:** 10.17113/ftb.60.03.22.7107

**Published:** 2022-09

**Authors:** Daniela Ilie, Andreea Iosageanu, Oana Craciunescu, Ana-Maria Seciu-Grama, Catalina Sanda, Florin Oancea

**Affiliations:** 1 National Institute of R&D for Biological Sciences, 296, Splaiul Independentei, 060031 Bucharest, Romania; 2 National Institute for R&D in Chemistry and Petrochemistry – Icechim, 202, Splaiul Independentei, 060021 Bucharest, Romania; § These authors contributed equally to this study

**Keywords:** fish peptides, proteinase K, antioxidant activity, cell migration, oxidative stress

## Abstract

**Research background:**

Various protocols for enzymatic hydrolysis of fish by-products are increasingly tested to ensure value-added products with functional and biological properties important for food, cosmetic and medical applications. In addition, they attempt to minimize waste from industrial processing and environmental requirements. This study aims to establish an efficient protocol based on two-step enzymatic hydrolysis of freshwater fish skin and to evaluate the effect of resulting bioactive peptides on free radical scavenging, redox balance and regulation of fibroblast proliferation and migration.

**Experimental approach:**

Pepsin-soluble collagen extracted from silver carp (*Hypophthalmichthys molitrix*) skin was hydrolyzed by proteinase K at specific sites under controlled conditions. The molecular mass of ultrafiltration permeate was determined by gradient electrophoresis and gel filtration chromatography. The biological activity of intermediate and small size bioactive peptides was evaluated in experimental models *in vitro* mimicking oxidative stress and skin wound conditions.

**Results and conclusions:**

Extracted fish collagen was hydrolysed using proteinase K, the most efficient enzyme for the cleavage of the primary structure of the molecule, as previously found *in silico*. Established optimal conditions increased the enzyme specificity and the process yield. Bioactive peptides exerted significantly higher scavenging activity on free stable radicals and hydroxyl radicals often found *in vivo*, compared to fish collagen. They stimulated fibroblast metabolism in a dose-dependent manner and up-regulated cell migration in a scratch wound model. Pretreatment of fibroblasts with induced oxidative stress using optimal concentrations of fish peptides prevented the increase of reactive oxygen species production. In conclusion, bioactive peptides from carp skin demonstrated valuable properties of maintaining redox balance and skin wound healing process improvement, which indicated further potential applications in the development of pharmaceutical and nutraceutical formulations.

**Novelty and scientific contribution:**

In this study the enzymatic hydrolysis was applied to isolated protein, in contrast to previous studies using waste tissue with variable composition. Recovered bioactive peptides acted not only as antioxidant agents, but also as regulators of oxidative stress and wound healing processes in skin cell models. Their nutritional and cosmetic application is recommended in novel formulations fighting skin ageing phenomena.

## INTRODUCTION

The nutritional value of fish by-products is almost identical to that of the edible parts so, nowadays, their reprocessing can lead to low-cost, value-added food products with improved properties (*1*). Among the high-grade products obtained by bioconversion of fish waste, bioactive peptides are important for their applications as alternative ingredients in functional food, nutraceuticals and pharmaceuticals, to prevent diseases and improve human health (*2*). As natural products with antioxidant activity, fish bioactive peptides can be used in food industry as an alternative to synthetic antioxidants (butylated hydroxyanisole - BHA, butylated hydroxytoluene - BHT), which may have adverse effects on enzyme activity and DNA (*3*). Fish peptides are also recommended as ingredients in cosmetic industry owing to their antioxidant activity, cryoprotective, photoprotective and moisture-retention abilities (*4*). Antihypertensive potential through inhibition of the angiotensin-converting enzyme (ACE) and their anticoagulant, antimicrobial and immunomodulatory activities recommend them as high-value active ingredients for medical applications (*5*, *6*).

Besides the naturally occurring bioactive peptides, they can be produced by enzymatic hydrolysis of fish protein or by-products to smaller fragments, usually consisting of 2−20 amino acid residues. Various biotechnologies have been proposed, at lab and pilot level, based on acid or alkaline hydrolysis of fish proteins, but final hydrolysates had low nutritional value due to degradation of essential amino acids, like Met, Trp, Thr, Lys, His and Ile, and were valorized as fertilizers (*7*). Enzymatic hydrolysis used various commercial proteases to obtain hydrolysates rich in valuable biologically active compounds, based on controlled hydrolysis degree under specific reaction conditions, according to the particularities of each enzyme type, *e.g.* pH, structure, specificity (*8*). Neutral enzymes of microbial origin (Alcalase, Flavourzyme) proved to be highly efficient in protein hydrolysis, as well as stable at different pH and temperature, but they were mainly applied to waste tissues with variable content of protein (*9*). Sequential or combined enzymatic digestion of fish by-products was performed in a bioreactor system followed by ultrafiltration, in order to separate functional peptides according to their molecular mass (*10*). Preliminary treatment is needed in case of fish by-product hydrolysis to avoid browning, unpleasant taste and odour of the final product, or formation of toxic compounds due to lipid oxidation (*7*).

To date, numerous marine sources (sardines, tuna, Alaska pollock, Nile tilapia, sea bream or leatherjacket) have been documented for valorization of huge amounts of by-products (skin, scales, bones and viscera) in the form of bioactive peptides (*11*, *12*). Still, few data exist on the production of bioactive peptides from by-products of aquaculture fish species, in particular from large Cyprinidae, like common carp (*Cyprinus carpio*), bighead carp (*Arystichtys nobilis*), grass carp (*Ctenopharyngodon idella*) and silver carp (*Hypophthalmichthys molitrix*) by neutral protease action. A biotechnology was proposed based on bacterial protease hydrolysis directly applied to grass carp skin, yielding a fraction of peptides with *M*<1000 Da that favoured *S. thermophilus* growth and the development of functional foods with probiotics (*13*). Hydrolysis of silver carp protein with Flavourzyme facilitated the production of peptides with negatively charged hydrophilic acidic amino acids, like aspartic and glutamic acids, for developing antibacterial agents used in food industry (*14*). Good recovery of low molecular mass peptides was obtained by Alcalase treatment of silver carp protein and they played an important role in metal chelation and inhibition of linoleic acid peroxidation (*15*). A study compared proteinase K and other treatments with endopeptidases applied directly to grass carp skin and reported low degree of hydrolysis, but different working conditions were used for each enzyme and their specific activity was not provided (*16*). High yield of bioactive peptides with low molecular mass was obtained by silver carp skin gelatin hydrolysis with collagenase or alkaline proteinase and applied for osteoporosis treatment in aged mice (*17*). According to *in silico* simulation, proteinase K and papain found numerous sites of action on collagen type I molecule and were the most effective for the production of small peptides by proteolysis, but the studies included only the primary linear structure (*18*). The functional (solubility, water retention, foaming, emulsifying) and biological (antioxidant, antihypertensive, antiproliferative) properties of bioactive peptides were found in significant correlation with their amino acid type and sequence, but also with their size (*19*, *20*).

The aim of this study is to characterize bioactive peptides obtained by proteinase K-assisted hydrolysis of collagen extracted from freshwater silver carp skin and then to evaluate their free radical scavenging potential, their effect on reactive oxygen species (ROS) production and cell migration in experimental models of fibroblast culture mimicking the oxidative stress and skin wound conditions.

## MATERIALS AND METHODS

### Materials

Silver carp skin was delivered on ice by a local fishery (Tulcea, Romania). Pepsin (EC 3.4.23.1), proteinase K (EC 3.4.21.64), Sephadex G-75, bicinchoninic acid (BCA) 2,4,6-trinitrobenzenesulfonic acid (TNBS), 2,2’-azino’-bis(3-ethylbenzothiazoline-6-sulfonic acid) (ABTS) and 1,1-diphenyl-2-picrylhydrazyl (DPPH) were purchased from Sigma-Aldrich, Merck (Taufkirchen, Germany). All other chemicals of analytical grade used in experiments were purchased from Sigma-Aldrich, Merck, unless specified otherwise. A culture cell line of mouse fibroblasts NCTC clone L929 and European Collection of Authenticated Cell Cultures (ECACC) maintained on Eagle’s minimum essential medium (MEM) were purchased from Sigma-Aldrich, Merck.

### Extraction of carp skin collagen

Collagen was extracted from silver carp skin using the enzymatic method with pepsin in acetic acid according to a previously described protocol (*21*). Briefly, skin was thoroughly washed in cold tap water, minced and extracted in 0.5 M acetic acid containing pepsin (2500 U/mg), in an enzyme/tissue mass ratio 1:10, at 4 °C, with gentle stirring, for 24 h. The process was repeated twice. The gel was separated from the undigested tissue, purified by precipitation with 0.7 M NaCl, centrifuged at 5000×*g* for 20 min and dialyzed against distilled water. The resulting carp skin collagen gel mass fraction was 1.2% with pH=5.5 and it was stored at -18 °C for 2 months.

### Enzymatic preparation of collagen peptides

**Fig. 1:**
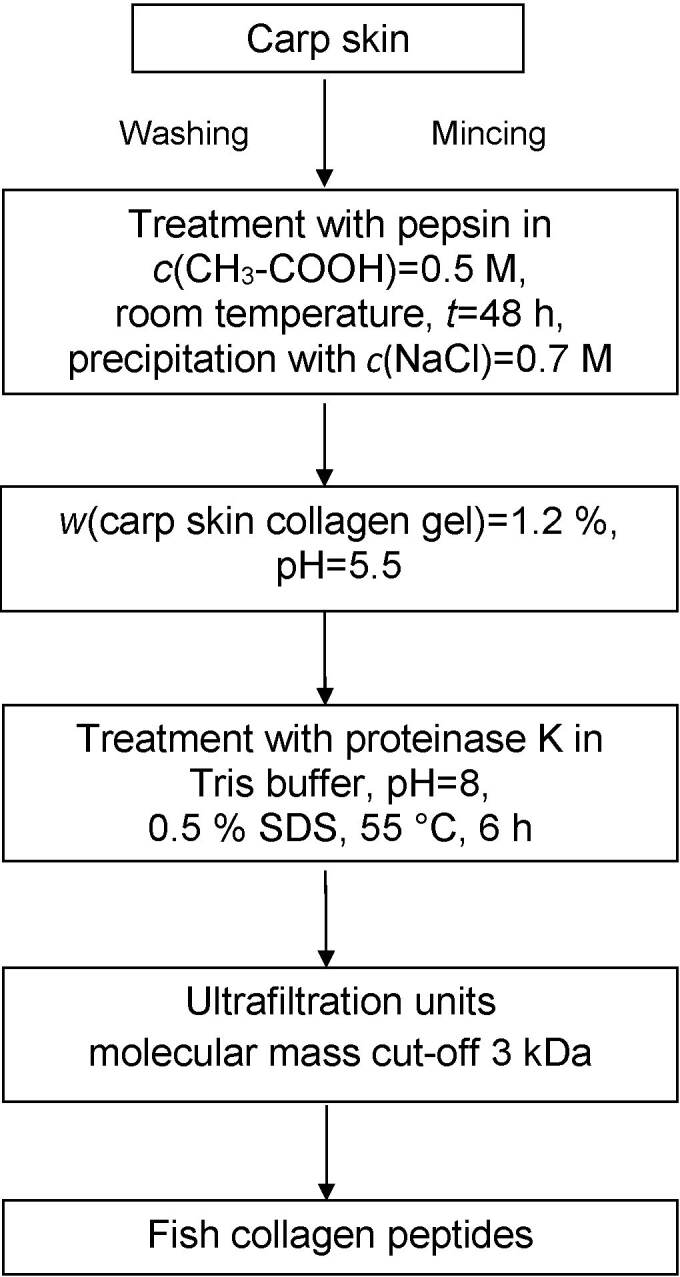
Scheme of two-step preparation of carp skin collagen by pepsin treatment and carp skin collagen peptides by enzymatic hydrolysis with proteinase K under controlled conditions

Enzymatic hydrolysis of carp skin collagen was carried out under specific conditions of pH and temperature (Fig. 1). The treatment protocol consisted of incubation of diluted carp skin collagen in 0.05 M Tris buffer, pH=8, containing 0.15 M NaCl and 5 mM CaCl_2_ at 45 °C in a water bath for 30 min. Then, proteinase K (10 U/mL) in the same buffer, pH=8, supplemented with 0.5% sodium dodecyl sulfate (SDS), as enzyme activator, was added and incubation continued at 55 °C for 6 h. The pH of the reaction mixture was maintained at an optimum value of 8 during incubation. Finally, the solution was heated at 95 °C for 5 min to inactivate the enzyme, then cooled at room temperature and centrifuged at 8000×*g* and 4 °C for 15 min. The supernatant representing the enzymatic preparation of carp skin collagen was subjected to centrifugal ultrafiltration using units with cellulose membrane at a molecular mass cut-off of 3 kDa (Amicon, Taufkirchen, Germany). Thepermeate containing carp skin collagen peptides was collected and stored at -18 °C until analysis. Each experiment was performed on 10 samples of carp skin collagen. All samples were analyzed for protein content by BCA assay and a standard curve was built with pig skin gelatin in the range of 0.1-3 mg/mL in order to calculate the process yield. Hydroxyproline (Hyp) content was determined in alkaline hydrolysate using chloramine-T and Ehrlich’s reagent, as previously described (*22*).

### Determination of the degree of hydrolysis

The concentration of free ɛ-amino groups was determined using TNBS assay, as previously described (*23*), recording the absorbance (*A*) of the resulting complex at 346 nm using a UV-Vis spectrophotometer V650 (Jasco, Tokyo, Japan). The degree of hydrolysis (DH) was calculated as a percentage relative to total hydrolysate prepared by carp skin collagen (CSC) incubation in 6 M HCl at 110 °C for 18 h, using the following equation:

DH=((*A*_sample_–*A*_CSC_)/(*A*_total hydrolysate_–*A*_CSC_))·100 /1/

### SDS-polyacrylamide electrophoresis

Carp skin collagen obtained by pepsin hydrolysis was analyzed by SDS-polyacrylamide electrophoresis, according to the method of Laemmli (*24*). The sample was denatured at 50 °C for 30 min and loaded on 7.5% SDS-polyacrylamide (PAA) resolving gel provided with 5% stacking gel. Migration was carried out at 30 V for 4 h using a Biometra source (Jena, Germany). A sample of rat tail tendon collagen was migrated under the same conditions. The high molecular mass marker (55−250 kDa) served to estimate the molecular mass of separated bands based on their retention factor (Rf).

The enzymatic preparations of carp skin collagen and the carp skin collagen peptides were denatured at 37 °C for 5 min and migrated in 10-20% gradient tricine SDS-PAA gel (Invitrogen, Waltham, MA, USA) at 90 V for 2.5 h (*25*). Low molecular mass marker (1.7−40 kDa) was migrated under the same conditions. The migration patterns were visualized by gel staining with Coomassie Brilliant Blue.

### Gel filtration chromatography

Gel filtration chromatography of the enzymatic preparations of the carp skin collagen and the carp skin collagen peptides was performed on a glass column (230 mm×15 mm) packed with swollen Sephadex G-75 gel, equilibrated with distilled water, as previously described (*26*). Samples (2 mg/mL) were loaded, eluted at a flow rate of 0.1 mL/min and monitored at 220 nm using a UV-Vis spectrophotometer V-650 (Jasco). Bovine serum albumin (66 000 Da), cytochrome c (12 384 Da), insulin (5777 Da) and bacitracin (1423 Da) were used as standards to build a molecular mass calibration curve for the calculation of the molecular mass of separated fractions.

### Determination of the antioxidant activity

#### ABTS assay

The scavenging potential of carp skin collagen peptides against ABTS radicals was evaluated as previously described (*27*). Briefly, the stock solution of 7 mM ABTS containing 2.45 mM potassium persulfate was diluted to reach a value of 0.70±0.02 at 734 nm (control). Diluted samples of carp skin collagen peptides (100 µL) were incubated with ABTS reagent (1 mL) at room temperature, in the dark, for 10 min. Then, the absorbance of the reaction mixtures was recorded at 734 nm with a UV-Vis spectrophotometer V-650 (Jasco). The percentage of ABTS radical inhibition was calculated using the following equation:

ABTS inhibition=((*A*_control_–*A*_sample_)/*A*_control_)·100 /2/

A calibration curve was built using Trolox, an analogue of vitamin E with known antioxidant activity, in the range of 0−150 µM. The antioxidant activity was expressed in µmol Trolox equivalents (TE) per g protein.

#### DPPH assay

A colorimetric assay was used to evaluate the scavenging potential of carp skin collagen peptides against DPPH radicals, as previously described (*28*). Briefly, freshly prepared solution of 0.25 mM DPPH (1.35 mL) was mixed with 0.1 M Tris buffer, pH=7.4 (0.9 mL) and carp skin collagen peptide sample (0.15 mL) at room temperature, in the dark, for 30 min. Then, the absorbance of the reaction mixtures was recorded at 517 nm using a UV-Vis spectrophotometer V-650 (Jasco). The percentage of DPPH radical inhibition was calculated using the following equation:

DPPH inhibition=((*A*_control_–*A*_sample_)/*A*_control_)·100 /3/

A calibration curve was built using Trolox in the range of 0−100 µM. The antioxidant activity was expressed in µmol TE per g protein.

#### Hydroxyl radical scavenging

The scavenging activity of hydroxyl (^•^OH) radicals generated by Fenton reaction was determined as previously described (*26*). Diluted sample of carp skin collagen peptides (80 µL) was mixed with 2 mM 1,10-phenanthroline (40 µL) in a 96-well microplate. Then, 40 µL of each 2 mM FeSO_4_ solution and *φ*(H_2_O_2_)=0.03% were added into the mixture. The microplate was incubated at 37 °C in the dark for 1 h and the absorbance of the resulting solution was measured at 536 nm using a Spectrostar nano microplate reader (BMG Labtech, Ortenberg, Germany). The percentage of ^•^OH radical inhibition was calculated using the following equation:

^•^OH inhibition=((*A*_sample_–*A*_negative control_)/(*A*_control_–*A*_negative control_))·100 /4/

where H_2_O_2_ was replaced by distilled water in control and the sample was replaced by distilled water in negative control.

The calibration curve was built using different concentrations of Trolox (0−500 µM). The antioxidant activity was expressed in mmol TE per g protein.

### Cell culture and cell viability assays

*In vitro* cytocompatibility of carp skin collagen peptides was evaluated according to the international standard ISO 10993-5:2009 (*29*) for medical devices by direct contact method. Briefly, mouse fibroblasts from L929 cell line were seeded in 96-well culture plate, at a cell density of 4·10^4^ cell/mL, and cultivated in MEM culture medium supplemented with 10% foetal bovine serum and 1% mixture of antibiotics. The plates were incubated at 37 °C in 5% CO_2_ humidified atmosphere. After 24 h, the medium was replaced with a fresh medium containing different concentrations of carp skin collagen peptides (10−400 µg/mL) and the incubation continued under standard conditions for 24 h. Cells cultivated in MEM served as negative control, while cells treated with 0.5 mM H_2_O_2_ served as positive control. Cells were also treated with 15 µg/mL ascorbic acid, a known agent for stimulation of fibroblast proliferation. Cell viability was evaluated by 3-(4,5-dimethylthiazol-2-yl)-2,5-diphenyltetrazolium bromide (MTT) assay, as previously described (*30*). The absorbance values read at 570 nm were proportional to the number of viable cells, which reduced MTT to formazan salt. Cell viability was expressed as percentage relative to control, considered 100%. The experiments were performed in triplicate.

Qualitative analysis consisted of light microscopy observations of cell morphology, under similar conditions as described above. Thus, L929 fibroblasts were cultivated in the presence of different concentrations of carp skin collagen peptides and incubated under standard conditions for 24 h. After medium harvesting, cells were washed, fixed in methanol and stained with Giemsa solution. Micrographs were acquired with an Axio Observer D1 optical microscope equipped with a digital camera (Carl Zeiss, Oberkochen, Germany).

### In vitro scratch-wound assay

The effect of carp skin collagen peptides on cell migration during wound healing has been evaluated in an experimental model of scratch-wound achieved in a monolayer of fibroblasts, as previously described (*31*). Briefly, L929 fibroblasts were cultivated in 35-mm Petri dishes in MEM culture medium until confluence. Then, a mechanical scratch was made on the monolayer using a pipette tip and fresh medium containing 100 µg/mL carp skin collagen peptides was added. Untreated cells served as negative control and cells treated with ascorbic acid (15 µg/mL) served as positive control. After 24 h of cultivation under standard conditions, cells were photographed using an AxioStar Plus phase contrast microscope (Carl Zeiss). The rate of wound repair was calculated after image analysis using ImageJ software (*32*). Thus, the density of migrated cells within the wounded area was calculated by automatic measurement of cells as particles in the captured images and then reported as percentage of the initial open area of the scratch.

### Determination of cellular ROS production

The effect of carp skin collagen peptides on ROS production was evaluated in an experimental model of oxidative stress induced in the fibroblast culture, as previously described (*33*). L929 cells were seeded in a 12-wells culture plate at a density of 5·10^4^ cell/mL and cultivated in MEM under standard conditions for 24 h. Adhered cells were incubated with fresh medium containing 100 µg/mL CSCP at 37 °C in humidified atmosphere with 5% CO_2_ for 24 h (pretreatment), and then the cells were treated with 50 µM *tert*-butyl hydroperoxide (*t*-BHP) for 30 min.

The assay used to measure ROS production at cellular level was based on the mechanism of hydrogen atom transfer using a cell-permeable fluorogenic probe 2’,7’-dichlorofluorescein diacetate (DCFH-DA). Upon reaction with free radicals, the formation of a fluorescent product was monitored. The cells treated as above were incubated with 10 µM DCFH-DA for 30 min and analyzed using a flow cytometer LSR II (Becton, Dickinson and Company, Franklin Lakes, NJ, USA). Acquired histograms were processed to calculate ROS production (%) in correlation with the fluorescence intensity using FlowJo v. 10.6.1 (*34*) and BD FACSDiva v. 6.1.3 (*35*) software. The cells incubated in normal medium and the cells pretreated with 12 µM ascorbic acid were processed under similar conditions and served as controls.

### Statistical analysis

All experiments were carried out in triplicate. The results were expressed as mean value±standard deviation (S.D.) for three experiments. Statistical analysis of the data was performed on each pair of interest using two-tailed, paired Student’s *t*-test. Differences were considered statistically significant at p<0.05.

## RESULTS AND DISCUSSION

In this study, a two-step enzymatic process was applied for the extraction of carp skin collagen, and then preparation of its peptides from carp skin residues by hydrolysis under controlled conditions (Fig. 1).

### Extraction and characterization of carp skin collagen

**Fig. 2:**
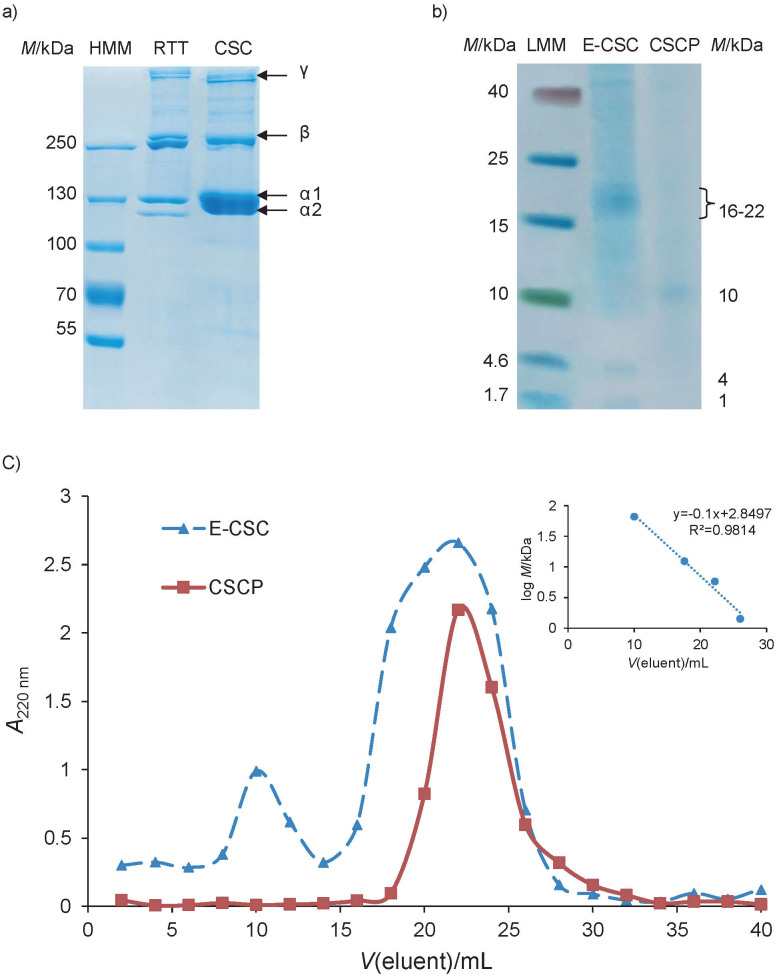
Electrophoretic migration patterns of: a) rat tail tendon (RTT) and carp skin collagen (CSC) in SDS-polyacrylamide gel showing the doublet characteristic pattern of α-chains, β-dimers and γ-trimers (arrows); b) enzyme hydrolysate of CSC (E-CSC) and carp skin collagen peptides (CSCP) in tricine SDS-polyacrylamide gel in gradient showing the corresponding molecular mass of visualised bands (arrows). High molecular mass (HMM) and low molecular mass (LMM) markers were migrated under similar conditions; and c) gel filtration chromatography of E-CSC and CSCP on Sephadex G-75 column. Insert presents the standard curve of molecular mass as a function of the elution volume

The first step was the extraction of undenatured collagen from fish skin using pepsin treatment under acidic conditions, as previously applied to mammalian tissues (*21*). After purification by salt precipitation, the pepsin-soluble collagen preparation with intact triple helix molecules was characterized by SDS-PAA electrophoresis under denaturing conditions (Fig. 2a). The pattern of carp skin collagen indicated the characteristic structure of type I collagen, consisting of three α chains, [(α1)_2_α2], similar to that of the collagen extracted, in a similar way, from rat tail tendon. The observed doublet, corresponding to two α1 chains and one α2 chain, had calculated molecular mass of 130 and 116 kDa, respectively. Typical bands corresponding to β-dimers and γ-trimers were also observed in both collagen extracts. Previous studies reported similar electrophoresis pattern of pepsin-soluble collagen from grass carp (*36*) and marine giant croaker (*37*). The minor variation of molecular mass values for α-, β- and γ-bands was observed within a narrow domain and correlated with the living environment (warm/cold water, salinity) and extraction method (acid, enzymatic), which could influence their structural characteristics (*37*). The chemical and structural characteristics of collagen extracted from fish species show their better rheological properties than mammalian collagen, which recommends it for cosmetic applications (*38*).

### Preparation and characterization of carp skin collagen peptides

The second step of peptide preparation consisted of carp skin collagen hydrolysis by proteinase K under specific and controlled conditions of pH, temperature and time of incubation in order to maximize the process. After a short incubation to denature its triple helical structure, the enzymatic hydrolysis of carp skin collagen followed the mechanism of action characteristic for an endopeptidase, cleaving the amino acid chain at a specific site containing peptide bonds adjacent to an aliphatic or aromatic amino acid. Thus, this enzymatic process of fish collagen hydrolysis was more controlled than a previously applied thermal treatment, which resulted in gelatin preparations with high MM (*39*).

The degree of carp skin collagen hydrolysis was 30.35% after proteinase K treatment for 6 h. Close values of 28.85 and 30.27% were obtained after 2 and 4 h of incubation, respectively, indicating that the enzymatic process reached a plateau. Previous studies have shown that the degree of hydrolysis varied between 11.50 and 33% for capelin protein hydrolysates and influenced their physicochemical properties and antioxidant activity (*40*).

After ultrafiltration, the obtained peptide preparation was preliminary characterized, showing an average of 87.5% protein, 8.6% hydroxyproline and 2.1% dry mass. The yield of peptide obtained from carp skin on dry mass basis of initial tissue using the two-step enzymatic process was 8%, a significantly increased value over previously reported 1.7−3.4% (*16*), indicating optimal hydrolysis parameters.

Fig. 2b presents the electrophoretic migration pattern of the enzymatic preparations of collagen, before and after ultrafiltration in tricine-SDS-gradient gel. Collagen preparation presented several bands distributed throughout the gel, indicating an efficient hydrolysis of collagen to fragments with *M*<40 kDa. Among them, a dense extended group of bands had calculated molecular mass corresponding to a range of 16−22 kDa and two narrow bands were observed at 4 and 1 kDa, respectively. Collagen peptide preparation obtained after ultrafiltration showed a more pronounced band at around 10 kDa, while faint blue coloured bands below 10 kDa indicated the presence of low molecular mass oligopeptides and peptides, considered bioactive compounds. Similar enzymatic hydrolysis method applied to grass carp skin using protease at 60 °C, pH=9, for 80 min resulted in the separation of a fraction containing peptides with *M*<1 kDa, which proved to be very useful for functional food development (*41*).

The enzymatic preparations of collagen and its peptides were also subjected to gel filtration chromatography in order to separate peptide fractions according to their molecular mass. Fig. 2c shows the chromatographic profile of samples obtained before and after ultrafiltration. The E-CSC preparation showed two distinct peaks, one corresponding to low elution volumes containing high molecular mass oligopeptides and proteins ranging between 28 and 112 kDa with a maximum intensity at 70 kDa. The second peak had higher intensity than the first peak, indicating high degree of CSC hydrolysis by proteinase K. CSCP preparation obtained after ultrafiltration presented a single peak, corresponding to peptides with a molecular mass ranging between 1.4 and 18 kDa with a maximum intensity at 4.5 kDa. This confirmed the separation of low molecular mass peptides with bioactive potential. Previous mathematical modelling showed that collagen was cleaved by proteinase K to small fragments of oligopeptides and bioactive peptides with low molecular mass, which could exert significant biomedical activity (*18*).

The collagen peptide preparation containing small and intermediate size peptides was further investigated for *in vitro* antioxidant activity and biological activity in cell culture experimental models.

### Effect of CSCP on free radical scavenging

The antioxidant capacity of low molecular mass peptide fraction was determined as scavenging potential against the free radicals ABTS, DPPH and ^•^OH, in comparison to that of the undenatured collagen (CSC). The results are given in Table 1. The scavenging potential of CSCP against free ABTS (68.6%) and DPPH (14.0%) radicals was moderate, compared to Trolox, the synthetic analogue of vitamin E. However, the values were significantly higher (p<0.05) than those of CSC (7.0 and 0.5%, respectively). Strong antiradical activity of collagen peptides incubated in the presence of ^•^OH radicals (71.0%) was recorded, representing highly encountered ROS within *in vivo* systems. Thus, our results showed that small peptides and oligopeptides were more efficient antioxidant agents than collagen, having more hydrogen atom- and unpaired electron-rich moieties to react with free stable radicals (*42*).

**Table 1:** Antioxidant activity of carp skin collagen (CSC) and carp skin collagen peptides (CSCP) determined as the capacity to scavenge free ABTS, DPPH and hydroxyl (^•^OH) radicals

Sample	ABTS assay			DPPH assay				^•^OH assay			
Inhibition/%	*b*(TE)/(µmol/ g)		Inhibition/%		*b*(TE)/(µmol/ g)		Inhibition/%		*b*(TE)/(mmol/ g)	
CSC	7.0±0.4	38.0±2.1	0.54±0.03		1.67±0.09		16.2±0.8		27.9±1.4	
CSCP	(68.6±3.3)*	(186.6±8.7)*	(14.0±1.2)*		(17.4±1.5)*		(71.0±4.0)*		(433.0±24.6)*	

The results are expressed as mean value±S.D. (*N*=3). Percentage of inhibition was determined at *γ*(CSC)=1 mg/mL. TE=Trolox equivalent, *p<0.05

Similar studies analyzed the free radical scavenging potential of fish bioactive peptides according to the fish species, enzyme used and hydrolysis parameters, showing different values between 30 and 90%, but higher than those of undenatured fish collagen (*43*). The great variability was also due to the wide spectrum of tested concentrations and assays, from ABTS, DPPH, ^•^OH and superoxide to lipid peroxidation or metal chelating capacity (*44*). Previous studies on papain hydrolysate of catfish skin collagen showed an increase of DPPH scavenging capacity in the first 2.5 h of hydrolysis, but lower value (63.06%) than of the hydrolysate of bone tissue (71.55%) (*45*). Fractions of marine fish oligopeptides with molecular mass between 5 and 10 kDa showed higher antioxidant activity against DPPH radicals (55%) than the fraction of low *M*<1 kDa peptides (37%) (*46*). The fractions with *M*>10 kDa had good antihypertensive activity due to the inhibition of ACE activity (*7*), while those found in grass carp skin hydrolysates with *M*<1 kDa (Gln-Pro, Trp-Pro-Pro) were good anti-ageing agents due to the ^•^OH radical scavenging and regulating redox processes (*47*). Also, Alcalase-treated silver carp muscle proteins containing low molecular mass peptides showed strong metal chelating capacity and lipid peroxidation inhibition (*15*). However, a recent study reported that no significant differences in ABTS scavenging potential were observed between peptide fractions separated according to molecular mass from the Alcalase-treated fish skin gelatin (*48*). No studies on the antioxidant activity of proteinase K fish hydrolysate were found.

As previously reported in databases storing collagen peptide sequences, proteinase K favoured the release of fish peptides with C-terminal hydrophobic amino acids and increased the number of amino and carboxyl groups in the collagen peptide preparation, favouring the free radical scavenging (*49*). In contrast, the long polymeric chains of fish skin collagen have limited functional groups, show abundance of hydrophobic amino acids (Ala, Gly, Pro, Val, Leu, Ile, Met, Phe) and a smaller proportion of hydrophilic amino acids with charged side chains (Glu, Asp, Arg, Lys) and polar amino acids (Hyp, Thr, Ser) than hydrogen bonds (*50*).

### Biological activity in cell culture

The effect of collagen peptides on the processes of skin wound healing and redox balance under oxidative stress conditions was investigated using *in vitro* experimental models developed in fibroblast culture. In this regard, the optimal concentration was first selected for testing.

### *In vitro* cytocompatibility

**Fig. 3:**
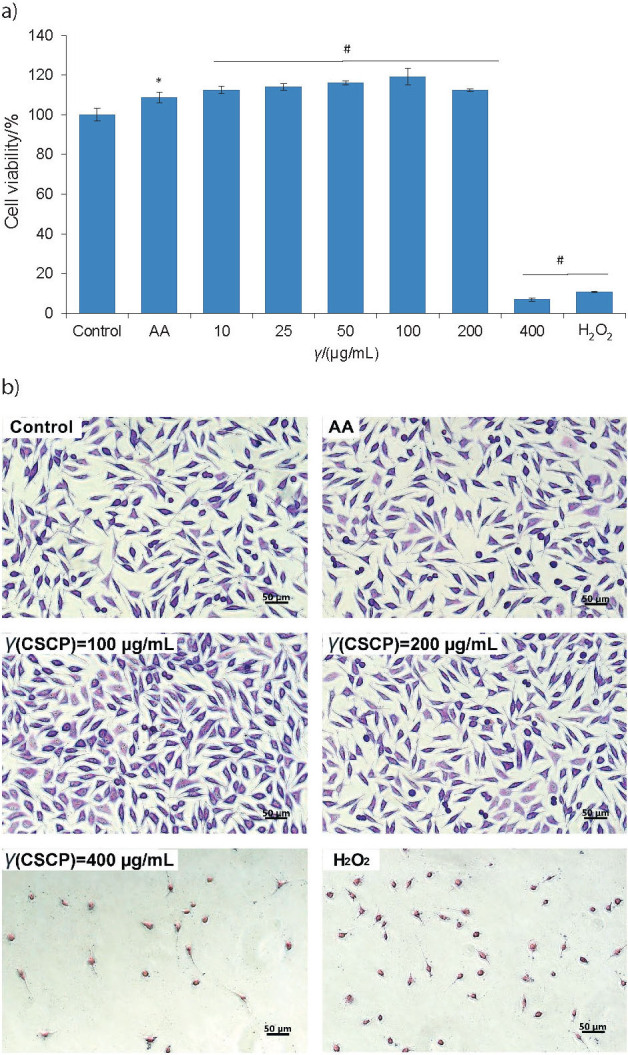
Cell viability of L929 fibroblasts cultivated in the presence of different concentrations of carp skin collagen peptides (CSCP) for 24 h, determined by: a) MTT assay, and b) light microscopy (Giemsa staining). Scale bar=50 µm. Untreated cells (negative control), cells treated with ascorbic acid (AA), as a regulator of cell proliferation, and cells treated with hydrogen peroxide (positive control) were cultivated under similar conditions. *p<0.05 compared to untreated cells (control), ^#^p<0.01 compared to control

Cell viability of fibroblasts cultivated in the presence of different concentrations of collagen peptides was evaluated by MTT assay. The results are presented in Fig. 3a. The values of fibroblast cell viability varied between 112 and 119%, in the range of peptide concentrations of 10−200 µg/mL. The maximum value of 119.2% was recorded at a concentration of 100 µg/mL peptides and it was close to that of ascorbic acid (108.7%). Higher concentrations of peptides significantly (p<0.05) decreased the cell viability as low as 6.8%, similar to hydrogen peroxide (10.5%), used as positive control. These data indicated that peptides were cytocompatible with fibroblast culture in a wide range of concentrations and had a similar effect to that of ascorbic acid, a known antioxidant agent involved in the upregulation of fibroblast proliferation.

The results of *in vitro* cytocompatibility were confirmed by light microscopy observations of cell morphology in fibroblasts cultured in the presence of the same carp skin collagen peptide concentrations. The acquired micrographs showed that peptide treatment maintained the normal cell morphology at concentrations between 10 and 200 µg/mL (Fig. 3b). The fibroblasts treated with carp skin collagen peptides had characteristic fusiform phenotype, euchromatic nuclei and clear cytoplasm, similar to that of untreated cells (control). Similar or higher cell density than in control culture was observed and the cells were homogeneously distributed in the culture plate. Cell treatment at higher peptide concentrations revealed morphological changes, degeneration and cell lysis, similar to the cells treated with hydrogen peroxide, while the cell density decreased.

Quantitative and qualitative data indicated that carp skin collagen peptides were cytocompatible with cultured fibroblasts at a concentration of 100 µg/mL and stimulated cell metabolism, and were subsequently tested in experimental models *in vitro*.

#### Effect of carp skin collagen peptides on wound healing

**Fig. 4:**
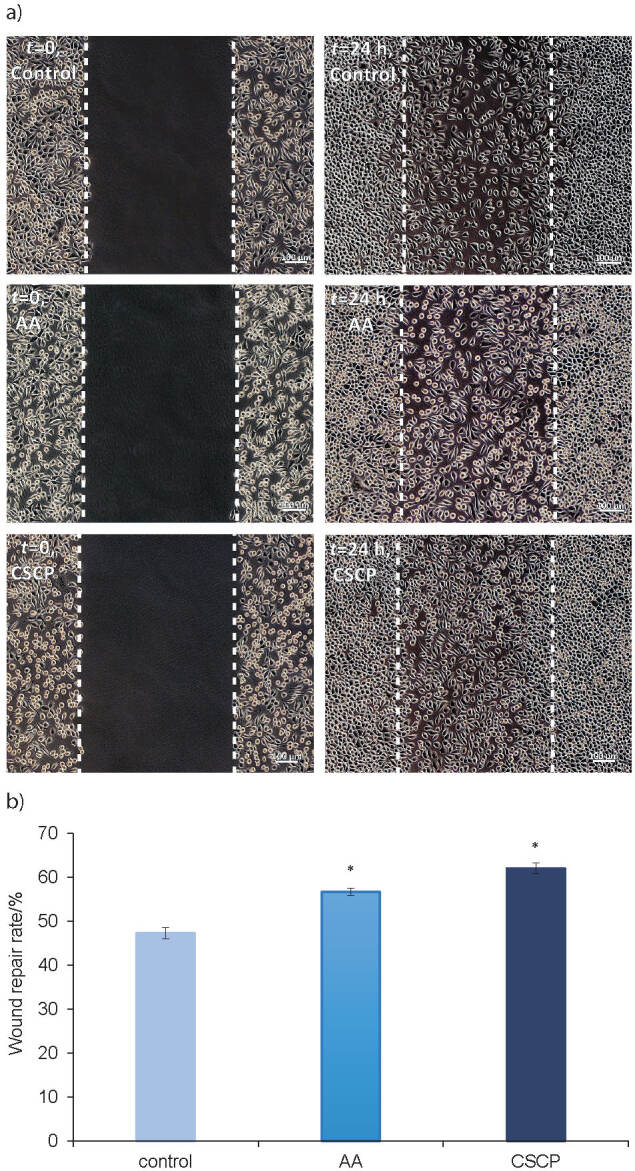
Wound healing assay of L929 fibroblasts in scratched monolayer of untreated (control), treated with ascorbic acid (AA) and carp skin collagen peptides (CSCP), at initial time (*t*=0 h) and after 24 h, observed by: a) phase contrast microscopy (scale bar=100 µm), and b) calculated as wound repair rate by image analysis using ImageJ software (*32*). *p<0.05 compared to untreated cells (control)

The effect of carp skin collagen peptides on cell migration was evaluated using an *in vitro* experimental model of scratch-wounded fibroblast monolayer. The results are given in Fig. 4. Phase contrast micrographs showed that the peptides induced cell migration to close the created wound after 24 h of cultivation, similar to the effect of ascorbic acid (Fig. 4a). Image analysis indicated that the scratch repair rate was significantly (p<0.05) higher in the fibroblasts treated with collagen peptides (62.1%) than in untreated cells (47.3%). The percentage was similar to that in ascorbic acid-treated cells (56.7%) (Fig. 4b).

These observations indicated a significant involvement of carp skin collagen peptides in the stimulation of fibroblast proliferation and active cell migration, as important phases during dermal wound healing and tissue remodelling. Previous studies showed that fish bioactive peptides had a low level of cytotoxicity in several fibroblast cell lines, indicating their potential for human health applications (*51*, *52*). Increased proliferation of NIH-3T3 mouse fibroblasts and human dermal fibroblasts was reported when using fish skin collagen peptides obtained by neutral protease hydrolysis, in accordance with our results (*4*, *53*). These data indicated that enzymatic hydrolysis of collagen favoured the increase of bioactivity. In previous studies, collagen-derived dipeptides, such as Pro-Hyp, were emphasized showing that oral ingestion of hydrolysates promoted fibroblast proliferation and wound healing (*54*). Also, collagen di- and tripeptides containing Hyp, such as Gly-Hyp, Pro-Hyp and Gly-Pro-Hyp, stimulated human fibroblast migration (*55*). Still, mixtures of fish collagen peptides can exert several activities as stimulators, mediators or cofactors of main growth factors and interact with cell membrane and cytoplasmic components, according to their size, structure, hydrophobicity and charge, as previously discussed (*10*, *56*). Alcalase hydrolysate of carp gelatin containing mainly 1−3 kDa peptides with good antioxidant activity was easily absorbed during mice gastrointestinal digestion, exerting biological effects of skin photoprotection (*57*).

#### Effect on redox balance at cellular level

**Fig. 5:**
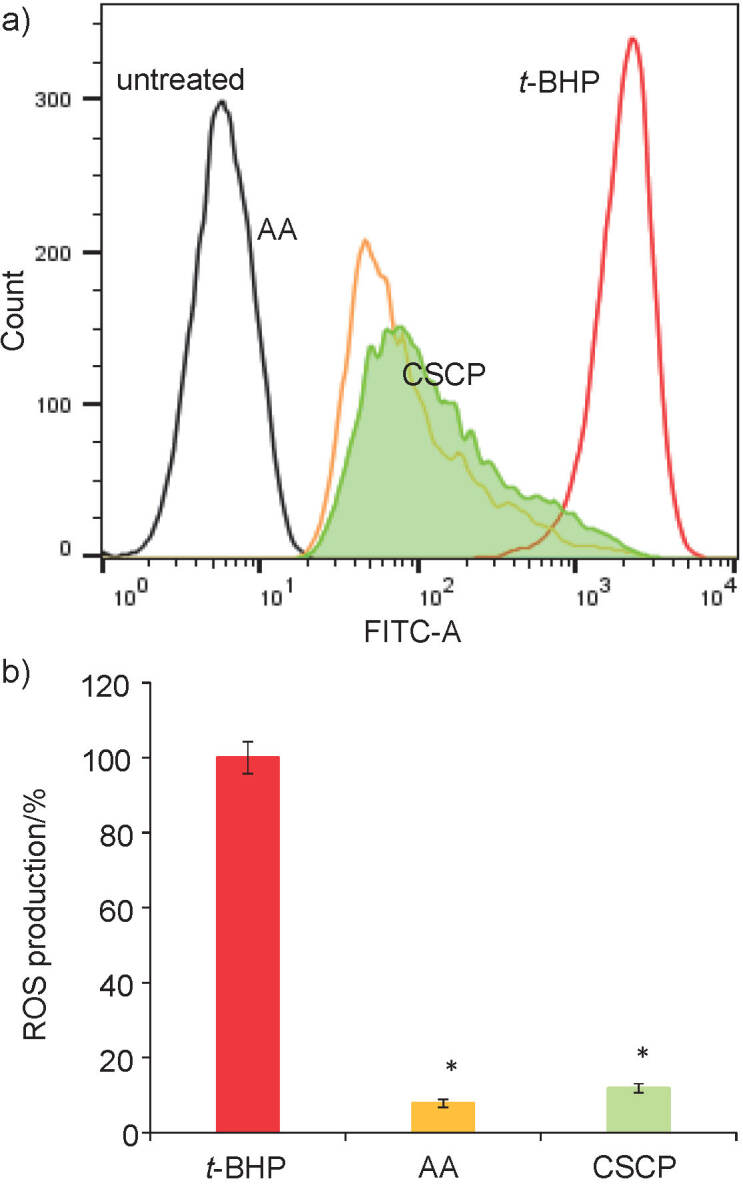
Intracellular reactive oxygen species (ROS) production measured by 2'-7'dichlorofluorescein diacetate (DCFH-DA) assay in untreated, *tert*-butyl hydroperoxide (*t*-BHP)-stressed and pretreated with carp skin collagen peptides (*γ*(CSCP)=100 µg/mL) and ascorbic acid (AA) L929 fibroblasts, presented as: a) fluorescence intensity using flow cytometry, and b) calculated as percentage of *t*-BHP-stressed cells using FACSDiva software (*35*). *p<0.05 compared to *t*-BHP-stressed cells

Involvement of fish protein hydrolysates in regulating the antioxidant factors at cellular level has lately been investigated (*20*). In our study, an experimental model of oxidative stress induced in the fibroblasts pretreated with carp skin collagen peptides was used to evaluate their effect on the cellular redox state and balance of ROS production by reaction with DCFH-DA and flow cytometry. The results are shown in Fig. 5. The histogram showed that stressed cells presented high level of fluorescence as the peak shifted to the right (Fig. 5a). Decrease of fluorescence was observed in the collagen peptide-pretreated cells and overlapped with the histogram of ascorbic acid-treated cells. Results of the analysis using FACSDiva software (*35*) confirmed a significant (p<0.05) decrease of ROS level down to 11.86% in the fibroblasts pretreated with CSCP and 7.81% in the cultures treated with ascorbic acid (Fig. 5b). All these data indicated an effective redox signalling of CSCP and ascorbic acid, used as antioxidant agent in food and cosmetic products, to achieve the balance between ROS production and scavenging.

Similar studies of the inhibition of ROS production in hydrogen peroxide-stressed macrophages revealed the antioxidant activity of collagen-derived peptides from marine sponge by trypsin hydrolysis (*58*). Oral administration for 60 days of products containing marine fish skin peptides supplemented with antioxidants increased plasma levels of oxidation markers (nitric oxide and malondialdehyde) in volunteers with aged facial skin, but within normal range, indicating no risk of oxidative damage (*59*).

## CONCLUSIONS

A new protocol was proposed for proteinase K hydrolysis of collagen extracted from silver carp skin that was efficiently carried out under controlled conditions and was followed by ultrafiltration to obtain a preparation consisting of intermediate and small size peptides with hydrophobic C-terminal amino acids due to enzyme specificity. The potential to scavenge free radicals was higher than that of fish collagen, in particular against ^•^OH radicals, also found in biological systems. Dose-dependent stimulation of fibroblast proliferation and up-regulation of cell migration indicated their functions in skin wound healing processes. Moreover, they prevented excessive ROS formation induced by oxidative stress in fibroblast cultures, maintaining the redox balance. Therefore, freshwater fish by-product processing yielded value-added peptides with potential to improve human health. Further studies will focus on structure-activity correlation and *in vivo* experiments in order to develop novel pharmaceutics and nutraceutics.
